# How Does Small-Pox Spread?

**Published:** 1901-11-09

**Authors:** 


### How does Small-pox Spread?

It is to be hoped—wished perhaps rather than to be fairly hoped for—that advantage will be taken of the present outbreak of small-pox in London to investigate the very important problem involved in the question, how does small-pox spread? We talk of infectious diseases, and we group under that head quite a considerable number of maladies, many of which a few years ago we should never have thought of regarding in that light. But while the number of diseases which we regard as infective is increasing, we are gradually attributing less and less importance to "infection," in the old sense of the term, as an intangible entity liable to attack anyone coming within reach of its malign influence. Typhoid fever we now attribute to a microbe; and although we may not know the vagaries of this creature outside the body, we do know, if we know anything, that it has to be swallowed by man before it can hurt him. Every year the infectivity of diphtheria is being more and more narrowed down to the passage of material particles containing germs from the patient that is to the patient that will be, and, instead of speaking of some vague and mysterious infection, we look to community in apples and slate pencils, to cups and spoons, to kissings and cuddlings, as the vehicles by which the disease is spread. Tuberculosis we pin down to dried expectoration; epidemic diarrhœa to an organism carried about and sown upon articles of food and drink: the story of malaria is now well known, and the transfer of yellow fever also is now regarded as the work of the mosquito. Plague is a disease of rats, and we look upon its transfer to man as in most cases the act of some biting insect. Puerperal fever we are no longer content to attribute to bad smells or insanitary surroundings; we trace it to an infection. Yet even that does not now satisfy us; we want to know how and by what means this infection is carried, and so we look to the hands and instruments of the doctor and the nurse. Pyæmia, which used to be thought to "infect" whole wards and attack whomsoever it listed, only wanting an open wound to serve as its point of entry, we now know to have been the result of dirty sponges, and now, under the influence of antiseptics, it has been so far banished that the younger generation of surgeons have hardly seen it. Cholera, the infection of which used to be regarded as a miasma creeping across the land in ghostly vapours—"cholera mists" as they were called—is now definitely pinned down to its own proper microbe and an epidemic of cholera becomes a mere phase in the natural history of the cholera bacillus. Everywhere we are finding out not only the microbe by which "infection" is caused, but the route by which it passes from victim to victim, and it is time that something of the same sort should be done by small-pox. We draw special attention to this matter because there is some suspicion abroad that for some time past attempts to strike out new lines on this particular subject are not looked on very favourably in official circles. The matter has been investigated by those connected with the Local Government Board; it has been decided that small-pox spreads through the air; the aërial diffusion of small-pox infection has become the official creed; thousands of pounds have been expended and much angry litigation undertaken in deference to it; and the subject is one which judicious medical officers of health pass by on the other side. Still it has to be faced. The investigations which led to the dogma of aërial diffusion or convection took place before we knew what we now know in regard to the part played by intermediate hosts in spreading disease; and although it may well be admitted that Mr. Power seemed to demonstrate with great exactitude the aërial convection of infection from acute cases of small-pox, we think that the time has arrived when the whole inquiry should be undertaken afresh.

At present we have two main lines of defence against small-pox, neither of which is altogether satisfactory. On the one hand we have general sanitary measures, which, useful as they are, are not enough to control small-pox. On the other hand we have vaccination, which, efficient as it is when properly applied, is by no means an ideal plan of combating any disease. Because we advise everyone in this generation to get vaccinated, and thus to protect themselves from small-pox, let no one imagine that we do not live in hope of a better way being found some time or another. How little we knew about malaria a few years ago, and how plain the whole matter now appears! How little we know about small-pox even now—so little that the most intelligent people of every nation see no better means of protection than the somewhat Irish method of having it in an attenuated form, for that is what vaccination comes to. Yet how much we might know, and how usefully that knowledge might be applied, if we could but discover the exact means by which the "infection" hops across from case to case. That is where we want to break the magic circle and to snap the chain, and we cannot but feel that the general acceptance of the theory of the aërial diffusion of small-pox infection as a final explanation of the facts stands in the way of progress.

